# Mental health and wellbeing implications of the COVID-19 quarantine for disabled and disadvantaged children and young people: evidence from a cross-cultural study in Zambia and Sierra Leone

**DOI:** 10.1186/s40359-021-00583-w

**Published:** 2021-05-15

**Authors:** Darren Sharpe, Mohsen Rajabi, Clement Chileshe, Sitali Mayamba Joseph, Ibrahim Sesay, James Williams, Siraj Sait

**Affiliations:** 1 Institute for Connected Communities (ICC), University of East London, Stratford Campus, Water Lane, London, E15 4LZ UK; 2 Department of Psychology, University of Tehran, Tehran, Iran; 3 Founder and President of Sport in Action (SIA), Lusaka, Zambia; 4 Department of Clinical Psychology, Kwame Nkrumah University, Kabwe, Zambia; 5 Philosophy in Physical Education and Sport, University of Zambia, Lusaka, Zambia; 6 Practical Tools Initiative, London, UK; 7 School of Business and Law, University of East London, London, UK

**Keywords:** COVID-19 pandemic, Children and young people mental health, Disabled, Disadvantaged, Zambia, Sierra Leone, Cross-cultural study, Low- and middle-income countries (LMICs)

## Abstract

**Background:**

The mental health impact of the COVID-19 pandemic and quarantining on children and young people (CYP) living in low- and middle-income countries (LMICs) has yet to be fully comprehended. CYP in LMICs are at utmost risk, given the COVID-19-related restrictions and social distancing measures, resulting in reduced access to school-based services for nutritional and mental health needs. This study examined mental health of CYP during the first COVID-19 lockdown in Zambia and Sierra Leone.

**Method:**

A total of 468 disabled and disadvantaged CYP aged 12 to 25 completed a planning tool that comprised the short Warwick-Edinburgh Mental Wellbeing Scale (SWEMWBS), as well as open-ended questions covering social connectedness, physical distancing and educational challenges during the lockdown. The community coaches screened individuals and families who could be eligible to receive emergency aid, and based on a convenience sample following distribution of aid, recipients were invited to complete the planning tool.

**Results:**

The data showed that participants in the global south have increasing anxieties and fears centred on accessing offline educational resources and income loss in the family effecting food security and their ability to return to education. Mean (SD) SWEMWBS scores for all participants in Zambia and Sierra Leone, were 19.61 (3.45) and 21.65 (2.84), respectively. Mental well-being scores were lower in females, children aged 12–14 and participants with two or more disabilities. Factors significantly associated with poor mental wellbeing in the sample were: type of disability, nationality, peer relationships, connection to others during the pandemic, knowledge about COVID-19, worry about the long-term impact of COVID-19, and the types of self-isolating.

**Conclusion:**

The study shows that participants who self-reported low levels of COVID-19 health literacy also scored low on the mental wellbeing self-assessment. Yet, despite undoubted limited resources, these CYP are doing well in identifying their needs and maintaining hope in the face of the problems associated with COVID-19 in countries where stigma persists around mental ill-health.

**Supplementary Information:**

The online version contains supplementary material available at 10.1186/s40359-021-00583-w.

## Introduction

In late December 2019, a novel coronavirus (Coronavirus disease, COVID-19) originating from Wuhan, the capital of Hubei Province in China, started to spread over many countries, and aroused global concerns [1]. As of the time this is written, globally, the number of confirmed cases and deaths attributed to COVID-19 reached over 140 million and 3,000,000 individuals, respectively [2]. Following Asian and European countries struggling with COVID-19, the COVID-19 pandemic was confirmed to have spread to Africa on 14 February 2020. The first confirmed case was reported from Egypt [3], and the first confirmed case in sub-Saharan Africa was in Nigeria [4]. Most of the identified imported cases arrived from Europe and the United States, rather than from China, where the virus started to spread. It is widely believed that there is substantial under-reporting in many African countries with less-developed healthcare systems [5]. Against this background, this paper focuses on the mental health and wellbeing of children and young people (CYP) during the COVID-19 lockdown in Zambia and Sierra Leone. In Zambia, the Ministry of Health reported its first two (2) positive cases of novel coronavirus on 18 March 2020. The two patients had travelled to France on holiday. From 17 March, the government had shut all educational institutions for an indefinite period and imposed some restrictions on foreign travel, local business and self-isolating behaviours [3]. On 16 March, the government in Sierra Leone banned public officials from travelling abroad and urged citizens to avoid foreign travel. Quarantine and severe restrictive measures were imposed for all passengers arriving from countries with more than 50 COVID-19 cases. On 24 March, President Julius Maada Bio announced a year-long ‘state of emergency’ in order to cope with a potential global pandemic. He confirmed the country’s first case of coronavirus on 31 March, contracted by a traveller from France on 16 March 2020 who had been in isolation.

Globally, to halt the COVID-19 outbreak and stop the rapid spread of the virus in the communities, governments instituted ‘social distancing’ measures and enacted nationwide or localised lockdown, travel restrictions, and limitations on the transport sector and industrial activities; as a result, many schools and universities have been closed [6, 7], and online-teaching and home-based learning strategies were begun, to keep students involved in educational institutions and classrooms [8]. CYP, like other groups of the population, have been affected by this pandemic [9], and they are most vulnerable to the drastic and unprecedented impact of it, as they are forced to study remotely and stay at home for a long period due to COVID-19-related measures and school closures, resulting in the least possible interpersonal interactions with classmates, alongside changes in sleeping patterns, unhealthy diets, too much screen exposure, and also insufficient physical activity [10–13]. Yet, tittle is known in populations from LMICs, or children with any type of disability or deprivation on their emotional well-being and coping strategies during COVID-19. It has been broadly represented that, compared to adults, the prolonged pandemic and its restrictions may lead to short-term as well as long-term physical, psychosocial, educational and mental health implications for children and young people [14–17]. In particular, vulnerable paediatric populations (including those who are struggling with homelessness, pre-existing psychiatric conditions, developmental disabilities, special educational needs (SENs), substance use and abuse, and domestic violence) necessitate more support through their family and healthcare services because of their noticeable mental and physical health multimorbidity [18–20]. The findings of research conducted during the pandemic on CYP with disabilities highlight drastic negative impacts of lockdown on varied aspects of their life, and difficulties for their caregivers [21–23]. For instance, stay-at-home policies, pressure of economic instability, and online learning strategies, along with the loss of many essential supports and rehabilitation services, have been the biggest challenges for parents of CYP with developmental disabilities [24, 25].

A range of vulnerability factors, such as social and ‘racial’ inequality, lack of access to health facilities and personal protective equipment, poverty level and being from economically disadvantaged backgrounds, special educational needs and learning disabilities, pre-existing mental health conditions, and knowledge about Coronavirus may determine the magnitude and scope of disproportionate impacts of the pandemic on this population. Roberton et al. [26] discuss that while it is less likely for COVID-19 to pose a threat to the health of CYP living in low- and middle-income countries (LMICs) immediately compared to older people, the long-term impacts of the pandemic and its mental health aftermath are of great concern [27]. Indeed, CYP in LMICs are at utmost risk, given the current restrictions and social distancing measures, resulting in reduced access to educational resources due to school closure, employment insecurity for their parents who operate as street vendors, and lack of access to school-based services for nutritional, mental and physical health needs.

## Background and context

To paraphrase the evolutionary psychologist Randolph Nesse [28], sometimes there are good reasons for bad feelings. Such is the case during COVID-19 lockdown. The emotional legacy of COVID-19 in CYP is likely to give rise to increased cases of panic disorders, general anxiety and social anxiety disorders. There are few healthcare systems in the global north which would be able to cope with the demand for post-COVID-19 mental health support for young people. In the case of Zambia and Sierra Leone, young people’s mental health needs have long been woefully under-researched and under-resourced [29, 30]. Yet still, CYP of African heritage are often denied a human face for their plight, and are instead characterised as being somehow genetically predisposed to emotionally cope with the intersecting trauma of civil unrest, disease and poverty. They need their voices heard, understanding and the right timely support. This makes good economic sense because most, if not all, mental health problems in adulthood start in childhood [31], and they will remain a persistent problem in Zambia and Sierra Leone, constituting a hidden burden of diseases if left unaddressed. Facing the current COVID-19 pandemic, mental health is thought to affect all young people, serving to exacerbate the social and structural determinants of health experienced by disadvantaged and disabled CYP. The UK Global Challenge Research Fund (GCRF) youth empowerment and community capacity building project was designed to empower disabled young people in the labour market and within their communities, but it was later repurposed to a Kick Out COVID-19 campaign in order to support CYP during the pandemic, specifically, disabled and disadvantaged CYP. The project distributed over 17,000 items of COVID-19 personal protective equipment (PPE) in Zambia and Sierra Leone, directly supported over 5,000 vulnerable and disadvantaged children, young people and families, and co-produced and disseminated public health messages on social media that have reached over 428,000 citizens in the global south, increasing their COVID-19 health literacy.

Research suggests that the greatest challenges in addressing young people’s mental healthcare in the global south are to do with legislation, human resource, stigma and infrastructure [32]. Dealing with some of these challenges may be helpful for these countries in addressing the effects of COVID-19, thereby reducing the increasing number of cases of mental illness. The Kick Out COVID-19 initiatives have also proven very productive in promoting mental health and wellbeing in individuals affected by the global pandemic. It is, however, noted from this research that mental health issues have been downgraded in Zambia and Sierra Leone, even as the situation is very much worsening. The economic and social situation is predicted to be even worse in Zambia and Sierra Leone than in the global north, due to the COVID-19 pandemic increasing the burden on weak healthcare systems and the human resources available in these countries. In the global north, there is growing recognition in the fields of social care, health and education that the psychosocial nature of CYP’s lives have changed, and that practice in relation to mental health protection and promotion is still trying to catch up with these changes. The changes relate to both higher levels of need resulting from chronic and severe mental disorder (schizophrenia, bipolar disorder, eating disorder etc.) and wider societal issues (e.g. high exposure to information communications technology, families living in poverty, unemployment, poor body image, radicalisation, anxiety and depression), which have an effect on receiving the right care, at the right time.

Research also suggests that CYP living in disadvantaged communities and/or living through a lot of adversity are more likely to experience lower degrees of social capital than their middle-class peers, as both a cause and effect of their set of circumstances. As a way of seeing this complex dynamic, this paper’s conceptual framework links human agency, resilience and capital as capacities, or processes, to explain how participants make choices, bounce back from wrong choice and adversities, and use their soft skills and social networks as a resource to support their mental health and wellbeing.

Zambian and Sierra Leonean CYP face all, if not most, of these challenges. These challenges take place in a context of current economic stagnation, gravely aggravated by the pandemic. From a perspective of economic growth, Zambia has encountered a number of setbacks in the past decade, which have contributed to straining its national budget. According to the African Development Bank (ADB), real Gross Domestic Product (GDP) growth slowed to an estimated 2% in 2019, down from 4% in 2018. Moreover, according to the updated IMF forecasts from April 2020, the outbreak of COVID-19 will cause a fall in GDP growth of -3.5% in 2020. To avoid stigmatisation of mental illness in the country, a lot must be done by conducting more research about the problem and well equipping the human resources within the country for handling more cases.

In Sierra Leone, young people under the age of 35 years comprise about 72% of the population. These young people are already facing challenges such as health inequalities, lack of access to education and skills training development, unemployment and underemployment. It is particularly important to present opportunities to establish a solid foundation for young people’s development and wellbeing. There are no existing services that provide tailor-made interventions for young people. Currently, there is no social support system for young people who are facing extreme poverty and vulnerability. Sierra Leone’s poverty is deeply entrenched, with an estimated 70% of the working population absorbed in the informal sector [33]. Poverty has escalated the risk of mental health problems, and this is compounded by disenchantment with a system that is characterised by social inequalities and poor economic and physical living conditions.

Mental ill health is considered to be a silent epidemic in Sierra Leone; a large proportion of the population are experiencing mental health issues as a result of various factors, including the 11-year civil war (1991–2002), the Ebola outbreak (2014–2015) and the mudslide (2017) [34–36]. The elements of social inequality, drug and substance abuse, and–more importantly–the current COVID-19 pandemic and its lockdown and restrictions on movement, have all posed serious threats to public mental health in the country, particularly amongst CYP, including those living with disabilities.

A survey conducted by the Ministry of Health and Sanitation and the World Health Organization [37], showed that about 700,000 people in Sierra Leone are suffering from serious mental health challenges and need medical attention. Of this figure, 350,000 have psychotic-related drug and alcohol abuse problems or illnesses such as cerebral malaria, more than 20,000 are suffering from bipolar manic depression disorder, and about 175,000 are experiencing epilepsy or schizophrenia. Most recently, Bah et al. [35] emphasised the seriousness of post-traumatic stress disorder (PTSD), psychosis and depression amongst victims of the civil war, Ebola and substance abuse. It has been estimated that 10% of the country’s population, including children and young people, are suffering from PTSD.

Correspondingly, the Zambia mental health country profile identifies that the high level of poverty in the country was already one of the major causes of mental health problems prior to the pandemic, especially for unemployed youth and people with disabilities [38]. The pandemic has had globally serious negative effects on the economy and on the labour market, and Zambia is no exception. Following the pandemic, mental health related problems for individuals and communities are expected to increase significantly, especially if no effective policies are implemented to assist the people affected by the lockdown.

The social and economic challenges for Zambia and Sierra Leone in tackling the growth of mental ill health in CYP concern legislation, human resource, stigma and infrastructure [32]. This paper does not have the space to address each in turn, and instead it focuses on the social challenge of COVID-19, thereby reducing the increasing number of COVID-19-related mental illnesses. Both research sites have paid little attention to the issue of childhood mental illness, which may impact these countries greatly, adding to the burden of disease.

There is an urgent need to investigate the negative impacts of prolonged quarantine in CYP with developmental disabilities and their families beyond the consequence of a viral infection. To our knowledge, there has been inadequate attention devoted to the mental, social and emotional needs of CYP with developmental disabilities during the COVID-19 mandatory confinement. More importantly, most of the current studies have been conducted in the global north and little attention has been paid to disabled and disadvantaged children and adolescents living in LMICs [23, 24, 26]. This study bridges this gap in knowledge to better understand the emerging mental and wellbeing needs disabilities and disadvantaged CYP during lockdown. The international importance of this study is illustrated in the ‘protective’ and ‘risk’ factors highlighted by participants, which originate from, but are not exclusive to, the fragile health systems and persistent vulnerability that participants face in their social settings. Ebola, civil conflict and natural disasters have provided important instructions on how CYP build resilience to better cope with such personal and social uncertainty caused by the pandemic. In a reversal of roles, the global south provides valuable lessons in the role that resilience and competencies play in CYP’s mental health and wellbeing. As social distancing restrictions loosen around the world, we will need to address the mental health and wellbeing challenge for CYP who have been seriously impacted by this pandemic.

## Methods

### Study design and participants

The participants were vulnerable children and young people (aged 12–25) (e.g. disadvantaged or living with a disability) living in the Northern and North West Provinces of Sierra Leone, and from the Central, Copperbelt, Eastern, Lusaka and Southern Provinces of Zambia, where each of the participating organisations have embedded community coaches.

### Survey content

The main instrument used in this study was a planning tool that comprised the short Warwick-Edinburgh Mental Wellbeing Scales, consisting of seven of the WEMWBS’s 14 statements about mental wellbeing (thoughts and feelings), which relate more to functioning than feelings, as well as demographic and open-ended questions covering social connectedness, physical distancing and activities during lockdown. Quantitative (yes/no and multiple-choice questions) and qualitative (open-ended questions) approaches were used comprehensively to analyse the data from the participants (see Table 1; copy of full survey in Additional file 1). After reviewing the literature on COVID-19 and social connectedness, main elements were developed by an international expert panel, including a child and adolescent psychiatrist, an educational psychologist, regional youth workers, and a sociologist, and applying the socioecological model. The study was approved by the University of East London (UEL) Research Ethics Committee, and it was launched on 21 June 2020 in both Zambia and Sierra Leone.

**Table 1:** Selected items used in the current analysis from the planning tool

Survey details	
Question	Response option
*A. Socio-demographics*	
Gender	Male
	Female
	Prefer not to say
Age	[dropdown, age rollup]
Nationality	Zambian
	Sierra Leonean
Zambia/Sierra Leone Province	[dropdown, provinces rollup]
Educational Level	[dropdown, specify]
Type of Disabilities	[dropdown, specify]
Family occupation/income per month	Very low (0$) to very high (1,3600$)
Who owns land/housing	[dropdown, specify]
B. *Wellbeing*	
I’ve been feeling optimistic about the future	None of the time (1)
I’ve been feeling useful	Rarely (2)
I’ve been feeling relaxed	Some of the time (3)
I’ve been dealing with problems well	Often (4)
I’ve been thinking clearly	All of the time (5)
I’ve been feeling close to other people	
I’ve been able to make up my own mind about things	
C. *Social networks*	
I feel I have lots of close friends who support me	one of the time
	Rarely
	Some of the time
	Often
	All of the time
I have a lot of friends and family around me who I can trust	
Since the outbreak of COVID-19, I have felt more connected to others	
D. *My response to COVID-19*	
We’d like to know how many people live in your household. Please tell us how many people, including yourself, are living with you:	I’m living alone
	2 people
	3 people
	4 people
	5 people
	6 people or more
How many rooms are in your home? (Not including any bathrooms or toilets)	[dropdown, number rollup]
Do you have access to outside space where you can currently play, walk or hang out?	Yes in public places/private place
	No
How would you rate your understanding to stay safe and well due to COVID-19 (Coronavirus)?	[Dropdown, specify]
Have you or other members of your family had COVID-19 (Coronavirus)?	[Dropdown, specify]
Are you worried about the long-term impact that COVID-19 (Coronavirus) and lockdown will have on your education, training and work?	[Dropdown, specify]
Which statement applies for you during epidemics of COVID-19 (Coronavirus)?	[Dropdown, specify]
Have you or any member of your family received any help response towards COVID-19?	[Dropdown, yes/no]
Do you feel you would need to be supported in relation to your response to COVID-19 and thereafter?	Not at all
	Yes, a bit
	Yes, a lot
	Yes, completely
What kind of supports would you like to receive in relation to your response to COVID-19 (Coronavirus) and isolation during epidemics and thereafter?	[Dropdown, specify]
Have you exercised during social distancing and taken part in sport?	[Dropdown, specify]
Is there anything else you would like to add about how COVID-19 has impacted on you and your life?	[Open-ended questions]
Suggest ways in which sports can be used as a solution to COVID-19	[Open-ended questions]

### Data collection

The community coaches screened individuals and families who could be eligible to receive emergency aid, and based on a convenience sample following distribution of aid (food, detergent and masks), recipients were invited to complete the planning tool. After reading an information sheet and the aim of the research, participants agreed to participate to submit their data.

### Outcome measures

Demographic data were collected, including sex, age (in five different groups), nationality, education level (primary schooling, secondary schooling, college, university, other), type of disabilities (physical, visual, hearing, mental health, intellectual, learning, two or more disabilities), household occupation/income per month (from $0 as very low to $1,360 as very high), and land/housing ownership (mother, father, brother, sister, grandmother, other).

Short Warwick-Edinburgh Mental Well-being Scale (SWEMWBS) [39]. Children and Adolescents mental wellbeing was measured utilising the shortened version of the Warwick-Edinburgh Mental Well-being Scale (WEMWBS). The SWEMWBS was designed to assess psychological functioning and emotional wellbeing. There are seven short statements starting with ‘I’ve been’, each with five response categories (1 = ‘none of the time’ to 5 = ‘all of the time’). The score range is 7–35, and higher scores indicate greater mental wellbeing. Categories for SWEMWBS were: ‘low’: 7–19.3; ‘medium’: 20.0–27.0; and ‘high’: 28.1–35. The SWEMWBS is a well-recognised measure for mental wellbeing and psychological functions, and it has been used in several countries. Acceptable psychometric properties, including construct, content and criterion validity, as well as internal consistency (α = 0.89), have been documented with a general population in the United Kingdom [39], as well as in many countries with different cultures, particularly in the African context [40–43].

### Statistical analysis

Of the 470 completed planning tools 2 participants did not pass the consistency checks (i.e. after cleaning the database for missing values, two cases were found as outliers). Sample characteristics were compared between individuals with and individuals without low mental wellbeing using chi-squared tests for categorical variables. SWEMWBS scores were compared between different sex and age groups. Effect sizes were estimated using Cramer’s V (chi-squared tests with categorical variables with more than two categories), Cohen’s d (t-tests with continuous variables), and eta squared (ANOVA with continuous variables). The association between several potential predictors (independent variables) and poor mental wellbeing (dependent variable) was studied using a linear regression model. Potential predictors included sex, age, education level, type of disability, income per month, social networks during self-isolation/social distancing, knowledge about COVID-19 and safety, and number of rooms and number of people in households. Results from the linear regression analyses are presented as lower and upper bound as well as p-value. The level of statistical significance was set at *P* < 0.05. All statistical analyses were performed in IBM SPSS 26 package [44].

## Results

**Fig. 1:**
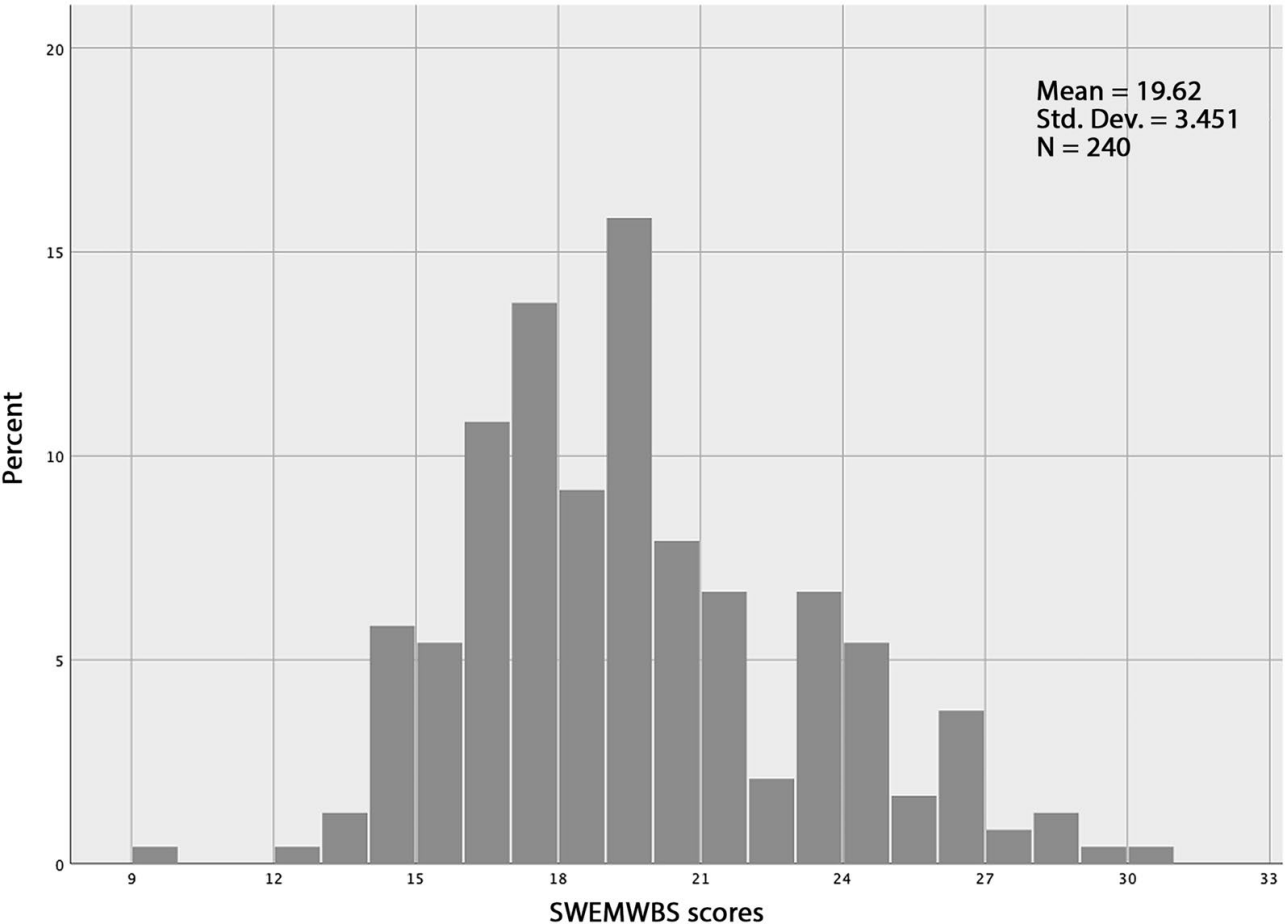
Unadjusted distributions of SWEMWBS scores in Zambia

**Fig. 2:**
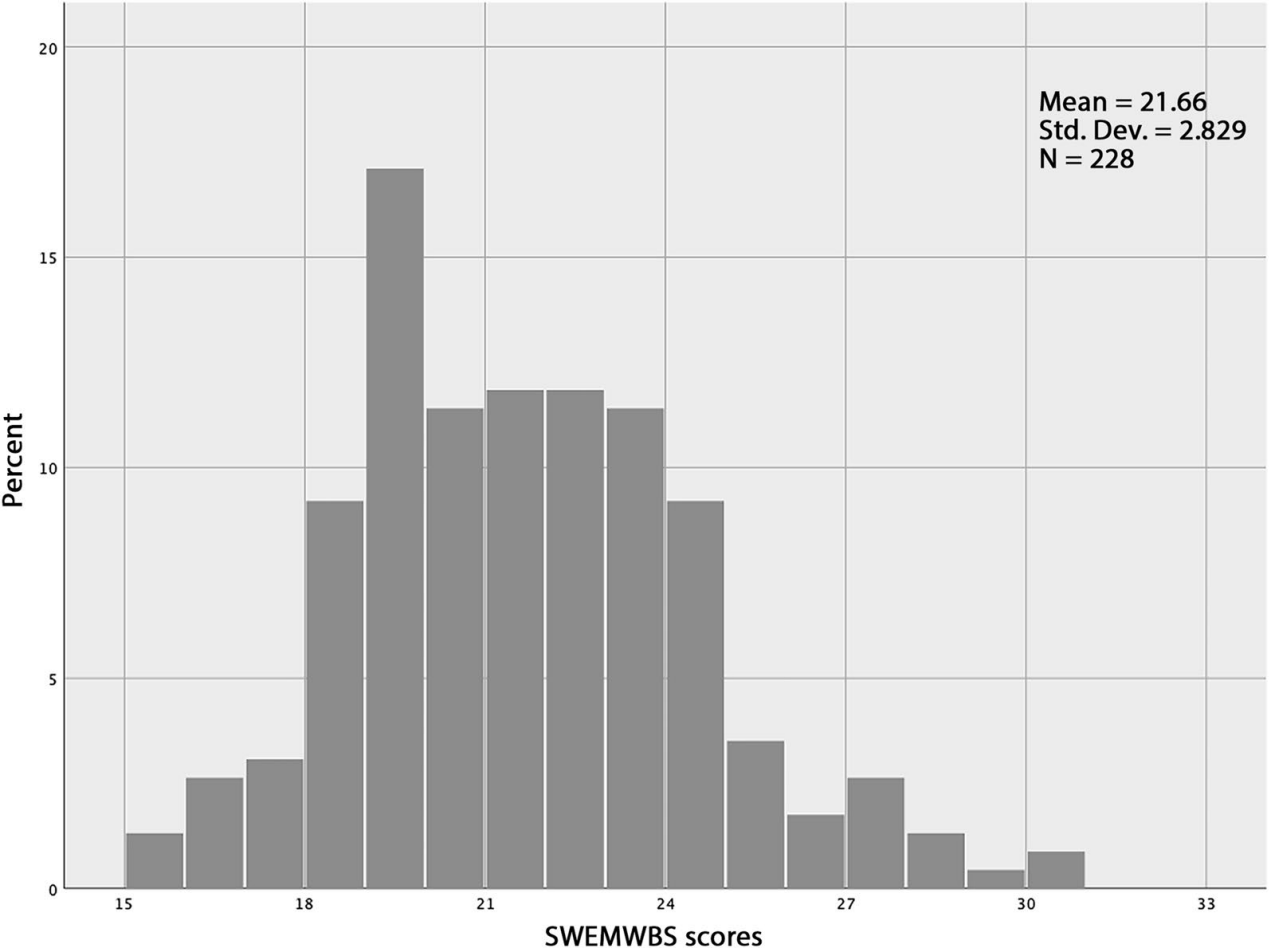
Unadjusted distributions of SWEMWBS scores in Sierra leone

This cross-cultural study included 468 participants aged 12 to 25. Of the whole sample, 57.9% were men and 42.1% were women; 44.3% of individuals were aged between 12 and 17 years (Table 2). Participants with poor mental health were more likely to be female. Mean (SD) SWEMWBS scores for all participants in Zambia and Sierra Leone, were 19.61 (3.45) and 21.65 (2.84), respectively (Table 3). Unadjusted distributions of SWEMWBS scores in Zambia and Sierra Leone are presented in Figs. 1 and 2, respectively. SWEMWBS scores were lower in women, younger children and participants with two or more disabilities. Finally, the results of the linear regression analyses are presented in Table 4. Factors significantly associated with poor mental wellbeing were type of disability (visual disability: β = − 0.887; *P* < 0.05; 95% CI − 1.763 to − 0.10; intellectual disability: β = − 0.888; *P* < 0.05; 95% CI − 1.771 to − 0.004; two or more disabilities: β = − 1.631; *P* < 0.001; 95% CI − 2.442 to − 0.821), nationality (Zambian: β = − 1.226; *P* < 0.001; 95% CI − 1.632 to − 0.821), close friends who support me (none of the time: β = − 0.949; *P* < 0.001; 95% CI − 1.569 to − 0.329; rarely: β = − 0.991; *P* < 0.001; 95% CI − 1.589 to − 0.393), connected to others during pandemic (none of the time: β = − 0.624; *P* < 0.05; 95% CI − 1.245 to − 0.002; rarely: β = − 1.303; *P* < 001; 95% CI − 2.025 to − 0.58; some of the time: β = − 0.7; *P* < 0.05; 95% CI − 1.327 to − 0.073; often: β = − 0.51; *P* < 0.001; 95% CI − 2.536 to − 0.484), knowledge about COVID-19 (poor: β = − 0.092; *P* < 0.05; 95% CI − 1.953 to − 0.232), worried about the long-term impact of COVID-19 (quite a lot: β = 1.042; *P* < 0.001; 95% CI 0.292 to 1.793), types of self-isolating (I am self-isolating as I am worried about spreading it to others: β = 1.268; *P* < 0.001; 95% CI 0.643 to 1.892; I am self-isolating to protect my family members, friends who have an existing medical condition: β = 1.031; *P* < 0.001; 95% CI 0.531 to 1.532).

**Table 2:** Participants’ distribution in terms of their socio-demographic characteristics

Characteristic	Category	Overall (%)	N = 468	Zambia	n	Sierra Leone	n
Sex	Male	57.9	271	31.6	148	26.2	123
	Female	42.1	197	19.6	92	22.4	105
Age	12–14 years	26.1	122	19.2	90	6.8	32
	15–17 years	18.2	85	9.8	46	8.3	39
	18–20 years	13.9	65	8.1	38	5.7	27
	21–23 years	10.3	48	3.2	15	7.0	33
	24–25 years	31.6	148	10.8	51	20.07	97
Education level	Primary schooling	35.3	165	27.5	129	7.6	36
	Secondary schooling	26.1	122	8.1	38	17.9	84
	College	0.6	3	0.0	0	0.6	3
	University	2.8	13	0.0	0	2.7	13
	Other	35.3	165	15.5	73	19.6	92
Type of disability	Physical disabilities	40.0	187	13.4	63	26.4	124
	Visual disabilities	4.5	21	4.4	21	0.0	0
	Hearing disabilities	7.3	34	7.2	34	0.0	0
	Mental health disabilities	4.7	22	4.7	22	0.0	0
	Intellectual disabilities	5.6	26	5.5	26	0.0	0
	Learning disabilities	1.9	9	1.9	9	0.0	0
	Two or more disabilities	12.0	56	11.9	56	0.0	0
	Prefer not to Say	24.1	113	1.9	9	22.2	104
Income per month	Very low	59.6	279	25.0	117	34.6	162
	Low	26.3	123	18.8	88	7.4	35
	Medium	14.1	66	7.4	35	6.6	31
	High	0.0	0	0.0	0	00.0	0
	Very high	0.0	0	0.0	0	00.0	0

**Table 3:** Mean and standard deviation scores for mental wellbeing (SWEMWBS) in the overall population

Characteristic	Category	Frequency (%)	Mean (Score)	SD (Score)	Effect size	*P* value
Sex	Male	271 (57.9%)	20.84	3.37	0.18	0.91
	Female	197 (42.1%)	20.29	3.22		
Age	12–14 years	122 (26.1%)	19.81	3.38	0.32	0.23
	15–17 years	85 (18.2%)	20.78	3.08		
	18–20 years	65 (13.9%)	19.85	3.05		
	21–23 years	48 (10.3%)	21.04	3.32		
	24–25 years	148 (31.5%)	21.35	3.34		
Education level	Primary Schooling	165 (35.3%)	19.87	3.27	0.25	0.058
	Secondary Schooling	122 (26.1%)	21.54	3.17		
	College	3 (0.6%)	25.45	2.48		
	University	13 (2.8%)	22.84	2.67		
	Other	165 (35.3%)	20.36	3.24		
Type of disability	Physical disabilities	187 (40.0%)	21.07	3.36	0.24	0.13
	Visual disabilities	21 (4.5%)	19.49	3.07		
	Hearing disabilities	34 (7.3%)	20.20	3.68		
	Mental health disabilities	22 (4.7%)	19.54	1.92		
	Intellectual Disabilities	26 (5.6%)	20.1	3.57		
	Learning Disabilities	9 (1.9%)	18.99	3.91		
	Two or More Disabilities	56 (12.0%)	18.55	3.37		
	Prefer Not to Say	113 (24.1%)	21.64	2.63		
Nationality	Zambian	240 (51.3%)	19.61	3.45	0.42	0.0
	Sierra Leonean	228 (48.7%)	21.65	2.84		

SWEMWBS Short Warwick Edinburgh Mental Wellbeing Scale. Poor mental wellbeing was defined as the presence of SWEMWBS metric score 7–19.3. This cut-off was selected based on previous literature. Lower SWEMWBS scores indicate poorer mental wellbeing

**Table 4:** Factors associated with poor mental wellbeing (SWEMWBS) in Zambia and Sierra Leone (N = 190)

Characteristics	Category	β	*P* value	t	95% CI	
					Lower bound	Upper bound
Sex		0.001	0.997	0.004	− 0.483	0.485
Age	12–14	− 0.68	0.054	− 1.942	− 1.371	0.011
	15–17	0.051	0.882	0.149	− 0.63	0.733
	18–20	− 0.083	0.808	− 0.243	− 0.76	0.593
	21–23	0.302	0.475	0.717	− 0.529	1.133
	24–25					
Education level	Primary schooling	− 0.221	0.451	− 0.755	− 0.799	0.357
	Secondary schooling	0.429	0.107	1.619	− 0.094	0.952
	College					
	University	1.07	0.947	0.067	− 30.487	32.638
Type of disability	Physical disabilities	− 0.518	0.074	− 1.8	− 1.086	0.050
	Visual disabilities	− 0.887	**0.047**	− 1.996	− 1.763	− 0.10
	Hearing disabilities	− 0.814	0.072	− 1.809	− 1.702	0.074
	Mental health disabilities	− 0.247	0.053	− 0.624	− 1.030	0.535
	Intellectual disabilities	− 0.888	**0.049**	− 1.983	− 1.771	− 0.004
	Learning disabilities	− 1.545	0.172	− 1.372	− 3.766	0.676
	Two or more disabilities	− 1.631	**0.0**	− 3.971	− 2.442	− 0.821
Income per month	Very Low	− 0.505	0.064	− 1.864	− 1.039	0.029
	Low	− 0.281	0.327	− 0.984	− 0.846	0.283
	Medium					
	High					
	Very high					
Nationality	Zambian	− 1.226	**0.0**	− 5.96	− 1.632	− 0.821
	Sierra Leonean					
Close friends who support me	None of the time	− 0.949	**0.003**	− 3.021	− 1.569	− 0.329
	Rarely	− 0.991	** < 0.001**	− 3.268	− 1.589	− 0.393
	Some of the time	− 0.48	0.105	− 1.629	− 1.062	0.102
	Often	− 0.782	0.113	− 1.59	− 1.751	0.188
Friends and family around me	None of the time	− 0.129	0.814	− 0.236	− 1.210	0.951
	Rarely	− 0.416	0.367	− 0.905	− 1.324	0.491
	Some of the time	0.201	0.618	0.499	− 0.593	0.994
	Often	0.485	0.303	1.032	− 0.442	1.412
Connected to others during pandemic	None of the time	− 0.624	**0.049**	− 1.98	− 1.245	− 0.002
	Rarely	− 1.303	**0.0**	− 3.557	− 2.025	− 0.58
	Some of the time	− 0.7	**0.029**	− 2.201	− 1.327	− 0.073
	Often	− 1.51	**0.004**	− 2.904	− 2.536	− 0.484
Household members	I'm living alone	1.213	0.94	0.076	− 30.357	− 32.783
	2 people	0.394	0.225	1.143	− 0.287	− 1.075
	3 people	0.376	0.216	1.243	− 0.221	0.974
	4 people	0.387	0.3	1.04	− 0.347	1.121
	5 people	− 0.361	0.436	− 0.781	− 1.273	0.551
Number of rooms	1 room	1.016	0.132	1.512	− 0.31	2.341
	2 rooms	0.722	0.288	1.065	− 0.616	2.059
	3 rooms	0.379	0.595	0.532	− 1.026	1.784
	4 rooms	0.132	0.853	0.186	− 1.273	1.537
	5 rooms	0.159	0.858	0.179	− 1.591	1.909
Access to outside space	Yes, in public places	− 0.122	0.676	− 0.418	− 0.698	0.454
	Yes, in my private space	0.914	**0.0**	3.47	0.394	1.433
Knowledge about COVID-19	Very poor	− 1.067	0.136	− 1.498	− 2.472	0.338
	Poor	− 1.092	**0.013**	− 2.504	− 1.953	− 0.232
	Moderate	− 0.664	0.085	− 1.734	− 1.42	0.092
	High	− 0.164	0.715	− 0.366	− 1.046	0.719
	Very High					
Worried about the long-term impact of COVID-19	Not at all	− 0.567	0.558	− 0.587	− 2.472	1.339
	Only a little	0.179	0.719	0.36	− 0.801	1.159
	Quite a lot	1.042	**0.007**	2.739	0.292	1.793
Types of self-isolating	I am living my life as normal and I don’t care about others	− 0.48	0.337	− 0.963	− 1.464	0.504
	l am self-isolating as I am worried about spreading it to others	1.268	**0.0**	4.003	0.643	1.892
	I am self-isolating to protect my family members, friends who have an existing medical condition	1.031	**0.0**	4.064	0.531	1.532
	I am self-isolating because I am not allowed to go out					
Exercise/sport during social distancing	Not at all	0.695	0.16	1.41	− 0.277	1.668
	No more than usual	0.975	0.052	1.956	− 0.008	1.957
	Rather more than usual	1.039	0.228	1.21	− 0.654	2.731

Values in bold text connote statistical significance, *P* < 0.05,* P* < 0.01, *P* < 0.001

Although most of the participants (72%) were self-isolating to protect their family members with medical conditions and/or so not to spread the virus, they broadly reported that they felt anxious and worried about the long-term impact that COVID-19 and lockdown will have on their education, training and work in the future. The study shows that nearly 91% of participants reported that they need to be supported considerably in relation to their response to the COVID-19 challenges and difficulties. Further analysis indicated that most of the participants (78%) would like to receive health and wellbeing workshops, support groups or seminars by ‘professions’ to tackle coronavirus and isolation during the pandemic and thereafter. They would also like to access online information about managing emotions and behaviour (12%), radio and television programmes (11%) and personalised online support from a professional or charities (7%) to increase their competencies to self-care.

Information and Communication Technology needs are superseded by a common concern over the prospect of future school clothing and fees, followed by an immediate need for basic educational equipment (e.g. schoolbooks and pens) and food insecurity (see Table 5).

**Table 5:** Participants’ concerns

Item	n	%
Food	20	18
Economic	40	37
Learning	36	33
Other	13	12
	109	100

Participant's accounts stress how their precarious educational careers have been worsened by COVID-19 restrictions. Participants remark:

My grandmother finds it difficult to fend for me and my brother, we need school fees, food and money for clothes. (Participant 11, aged 14)

I would like to be supported with textbooks, so that even when we are not going to school, I will have books to write and read from. (Participant 56, aged 12)

I need story books to help me improve on my reading, new clothes and education support. (Participant 450, aged 13).

I would like to receive sponsorship, learning materials, uniform and books to help me understand about school. (Participant 467, aged 17)

Financial support for acquiring basic needs or sponsorship to finish my secondary school. (Participant, 283, aged 15)

Need to have own shelter, we need food, I need to go back to school. (Participant 89, aged 15).

The participant’s accounts also highlight how finance, food and schooling concerns intersect and serve as the central risk factors undermining their ability to achieve stable mental health and wellbeing. Yet still, participants also identify the short-term protective factors they need. Participants comment:

More information on COVID-19 is needed as some people say there is no COVID-19. (Participant 316, aged 14)

Financial support for physiotherapy. (Participant 236, aged 21).

I need help with enrolling in a special needs school. (Participant 193, aged 13).

My mother sells on the street; she needs a permanent place for her business to take care of me and my school needs. (Participant 330, aged 15)

We need support in terms of food and some materials to prevent COVID-19. (Participant 421, aged 19)

## Discussion

In our study a planning tool was used to understand the subjective perceptions of social connectedness and physical distancing during lockdown (i.e. resilience and competency) and measure mental health problems (i.e. depression and anxiety) among 468 Zambian and Sierra Leone disabled and disadvantaged CYP in response to the COVID-19 pandemic. Illustrated in Table 5 are the young people’s self-defined ‘risk’ factors that should be addressed as part of the local response to combat the side effects of COVID-19. Demonstrated here are only the symptoms of the social and structural determinants of health made worse by the virus, but which have much deeper roots than the pandemic. A return to normalcy for the sample group will arguably provide little relief in achieving long-term stability in their mental health, if protective factors are not erected around them.

Despite the lack of access to, and availability of, community-based support services, the participants suggest a variety of different ways that they are managing their lockdown experience, and they also reveal a common set of concerns underlying their present situation and prospects. Unlike their peers in the global north, remote learning has not been a viable option during lockdown and being out of education has been a major source of anxiety for school age participants.

The planning tool respected CYP as active subjects rather than passive objects of study, which has enabled us to gather rich insights into the interior worlds of CYP that are contextualised and influenced by their social settings. The value of using subjective wellbeing as our central ontological gauge – rather than using a purely psychological wellbeing measurement tool – is that it has allowed for the cultural sensitivity for both the participants and researcher to gather and interpret personal information during a pandemic, the roots of which are socio-cultural, persistent and complex [45].

White and Eyber [45] highlight the “limitations of quantitative approaches, which are particularly clear when they are used in societies other than those in which they were designed, since they inevitably reproduce their own categories, and are unable to recognise understandings of the world that are different to their own “ [45]. For instance, economic indicators on happiness dominate discourse on wellbeing in the global north and exhibit a cultural bias towards mental wellbeing in the global south. We therefore used SWEMWBS, primarily because it has been tried and tested successfully in Zambia [41], in combination with open questions co-designed with our local partners that get to the roots of the problems. The involvement of local community coaches and youth champions in the design of the planning tool focused greater attention on the impacts of context, culture and local survival strategies on CYP’s responses to adversity.

Semo and Frissa [46] argue that the potential effect of COVID-19 on mental health in sub-Saharan Africa could be tremendous, given the weak healthcare systems. Like the Ebola epidemic of 2014–2016, COVID-19 is expected to cause anxiety, depression and post-traumatic stress disorders. Therefore, the study question shifted to learn more about the resilience and competencies exhibited by the participants to cope with COVID-19 restrictions. Resilience is a multifaceted capability, and our results show that participants who self-reported low levels of COVID-19 health literacy also scored low on the wellbeing self-assessment, whereas the majority of the participants faced personal and social challenges as a result of COVID-restrictions, but have been able to communicate and share their hopes for the future. The team at the Institute for Connected Communities (ICC) are working with local providers to scope out actions to map, unlock and mobilise community assets to better target limited resources to support the most vulnerable and disadvantaged, with immediate help centred on the risk factors that matter most to them (e.g. food security, at-home learning materials, and schooling). This is against the backdrop of weak or poorly resourced health and education infrastructures. For instance, Smith et al. [47] emphasise the high prevalence of inadequate hand washing practices in low-income countries linked to CYP experiencing severe food insecurity. They call for interventions that disseminate good hand-washing practices in children from low-to medium countries should not be dismissed.

Compellingly, the planning tool has identified that not all CYP are doing so well under COVID-19 restrictions, which is reflected in the SWEMWBS scores. Moreover, a minority of participants have distinguished the risk factors associated with the debasement in their subjective sense of mental wellbeing. Notably, participants living with visual impairments and/or with two or more disabilities have identified themselves as being most at risk of a deterioration in their mental wellbeing during lockdown. The recognition in a change of mindset/feeling has been given emphasis when there are no, or limited, friendship support networks open to participants, and being sometimes or rarely connected with ‘others’ during lockdown. In these situations, participants reported often feeling worried about the long-term impact of COVID-19, and despite having such feelings, they said that they stayed committed to self-isolation to prevent the spread of the virus and to help protect their families and friends. We now turn to look in more detail at how participants draw on their internal and external resilience to deal with change and uncertainty bought on by COVID-19 restrictions.

### Competencies

The decision-making competency of participants does not appear to have been impacted during the COVID-19 restrictions, despite significant information (e.g. educational and personal) on self-care being incomplete and/or ambiguous during lockdown. In this respect, the study was successful in uncovering the participants’ competency levels in two ways: participants showed themselves to be capable social and empirical agents through the effective engagement in the planning tool that was undertaken in a structured dialogue; and the participants in the study enjoyed the participative involvement in the projects, which modelled health-creation techniques and distributed food aid and masks during the lockdown period.

In the latter case, participants shared important insights into their mental health anxieties, which have been formed against the backdrop of pre-existing health conditions and precarious educational pathways strained under COVID-19 restrictions. Studies [48–50] from each of the research sites show how health inequalities and epistemic injustice compound issues faced by CYP for their emotional, social and physical development. This results from: a lack of early interventions for mental health and wellbeing; societal age-bias that privileges the views and decision-making of older people at the expense of the young; the negative effects of social stigma on families who might fail to marry off sons/daughters exhibiting visible problems; the entrenched role of religiosity (both Christian and traditional beliefs) that serves as both a diagnosis technique and treatment for mental ill health; the toxic dilemma of increasing exposure to Western lifestyles juxtaposed with limited local opportunities; the pressures of being young carers and/or economic contributors to the family and getting an education; and, finally, limited public health promotion to better build understanding of the causes and effects of childhood mental ill health. This is borne out in the number of 12–14-year-old and 24–25-year-old participants who very much experience social labelling and stigma rooted in local norms of behaviour and cultural values as they become social agents and/or transition into work. Failure to do so successfully places increased stress on the CYP, impacting their mental health and wellbeing.

### Resilience

The ways in which the participants in this study demonstrate their resilience (e.g. an ability to bounce back from the often emotionally stressful circumstances) are both divertive and reductive. Both active and passive divertive strategies can have reductive outcomes in terms of alleviating emotional stress as well as relief from boredom in often restrictive environments. For instance, participants typically lived in low-income households, in large-sized groupings, frequently overcrowded, but with access to plenty of outdoor space. Untypically in the global south to be confined to the dwelling, participants have reportedly remained optimistic, and have focused on continuing their education and sourcing learning materials, essential items such as mobility aids, and exploring ways to earn extra income [51]. López-Bueno et al. [52] hypothesis that long periods of free-movement restrictions may negatively affect cardiorespiratory fitness and health in CYP is not borne out in this study. The respondents’ prior optimism is unchanged and serves as a protective factor to their mental health and wellbeing. Whilst the emerging findings reflect the participants self-defined COVID-19 priorities during wave one, they also reflect the inherent challenges in the partial fulfilment of the national development plans [53] in each of the participating countries.

## Study limitations and future directions

Although our study provides valuable source of information about emerging mental health implications of the COVID-19 pandemic on disable and disadvantaged CYP living in LMICs, it is not without its limitations. Due to our convenience sampling strategy (i.e. which knowingly produced a selection bias in favour of participants in recipient of project aid) we inevitably increased the likelihood of recall bias amongst participants who possibly stressed personal deficits over assets in their self-report to increase their chance of on-going project aid. This was partially dealt with by the research team in the careful selection of the research questions, choosing an appropriate data collection method, and how questions were then asked to individuals. A second limitation to the study was the small sample size. Despite the good response rate across all the participating study sites our findings are context specific and may not all be generalisable to other neighbouring low-income countries. For future studies, it is recommended and necessary to investigate the long-term impacts of the COVID-19 pandemic and post-COVID-19 recovery for CYP living with developmental disabilities in LMICs.

## Conclusion

The findings mirror other studies illustrated by Theron [54], who concludes that the resilience of sub-Saharan CYP is a complex, social-ecological process supported by relational, personal, structural, cultural, and/or spiritual resilience-enablers that makes African pathways of CYP resilience distinctive. This is noted in a disregard for values or practices that could constrain resilience, which works both for and against CYP mental health climbing up the policy agenda during the COVID crisis (i.e. resources, capacities, engagement/implementation). What is urgently needed is a rationalised and planned mental health support strategy at a community level that unlocks and leverages access to good quality COVID support and also educational packages [55–57]. More research is needed to expand our knowledge and understanding about how to strengthen community health and education resources to support vulnerable and disadvantaged CYP at the earliest point possible. Addressing the unmet needs of CYP with complex health needs during COVID-19 has clear benefits for the individual and the community, and the consequence of doing nothing will have serious implications for the future development of Zambia and Sierra Leone.

## Supplementary Information

**Additional file 1**. Copy of the Kick Out COVID-19 survey.

## Data Availability

The data that support the findings of this study are available from the corresponding author, upon reasonable request.

## Abbreviations

CYP: Children and young people
COVID‑19: Coronavirus disease 2019
SWEMWBS: Short warwick-edinburgh mental wellbeing scale
PTSD: Post-traumatic stress disorder
GDP: Gross domestic product
LMICs: Low- and middle-income countries
WHO: World health organization
