# Supplementary material for: Mental health and wellbeing implications of the COVID-19 quarantine for disabled and disadvantaged children and young people: evidence from a cross-cultural study in Zambia and Sierra Leone
Source: BMC Psychol. 2021 May 15;9:79. doi: 10.1186/s40359-021-00583-w (PMC8123096; doi:10.1186/s40359-021-00583-w)
Supplement: Supplementary file 1 — Additional file 1. Copy of the Kick Out COVID-19 survey. [file 40359_2021_583_MOESM1_ESM.pdf]

Mental Health and Wellbeing Implications of the COVID-19 Quarantine for Disabled and Disadvantaged Children and Young People: Evidence from a Cross-cultural Study in Zambia and Sierra Leone

Darren Sharpe[1], Mohsen Rajabi[2], Clement Chileshe[3], Mayamba Sitali[4,5], Ibrahim Sesay[6,7], James Williams[7], Siraj Sait[8]

1. Institute for Connected Communities, University of East London, London, UK. D.sharpe@uel.ac.uk

2. Department of Psychology, University of Tehran, Tehran, Iran. Rajabimohsen@ut.ac.ir (https://orcid.org/0000-0002-0254-9027)

3. Founder and President of Sport In Action (SIA), Lusaka, Zambia. Clementchileshe@gmail.com

4. Department of Clinical Psychology, Kwame Nkrumah University, Kabwe, Zambia. Mayambasitali@yahoo.com

5. Philosophy in Physical Education and Sport, University of Zambia, Lusaka, Zambia.

6. Founder and Chief Executive Officer, Sierra Leone Diaspora Development Foundation, London, UK. I.sesay@uel.ac.uk

7. Practical Tools Initiative, London, UK. James@practicaltoolsinitiative.org

8. School of Business and Law, University of East London, UK. S.sait@uel.ac.uk

*Corresponding Author: Darren Sharpe

Institute for Connected Communities, University of East London, Stratford Campus, Water Lane, London, E15 4LZ, UK.

Email address: d.sharpe@uel.ac.uk

Telephone: +442082234325

Kick Out COVID-19 Planning Tool

Page 1: Introduction

Ethics Approval Reference: **ETH1920-0212**

General Information
The Kick Out COVID-19 study will tell us how children and young people are coping during the covid-19 (coronavirus) pandemic, and what support they will need coming out of lockdown. We hope this will help us to understand the needs of children and young people living with disabilities or socially disadvantaged.

Thank you for your interest in this online planning tool. You have been invited to take part because you are a child or young person living in Zambia or Sierra Leone. Please read through this information before agreeing to take part by ticking the 'yes' box below.

You may ask any questions before deciding to take part by contacting the researchers (Darren Sharpe, Institute of Health and Human Development at the University of East London; details below).

You will be asked to answer some questions about how you are coping during the Covid-19 pandemic. The questions relate to your family life and relationships, overall health and wellbeing, and what support you need now and in the future. The questions cover a small number of areas so that we can get an overview of how things are for you currently, which will help us better understand what kind of support you might need later.

You don't need any background knowledge and there are no right or wrong answers. The planning tool should take around 10-15 minutes and you can take a break and come back to it if you wish.

The information you give us will be analysed by academic researchers at the University of East London and other institutions that have been approved by the research team so that we can work out the best way to support children and young people.

Do I have to take part?

Please note, taking part is voluntary. If you do decide to take part, you may withdraw at any point during the planning tool for any reason before submitting your answers by pressing the ‘Exit’ button/closing the browser.

How will my data be used?

Your answers will be anonymous, and we will take all reasonable steps to make sure that they remain confidential. Your email address will be removed from the rest of the answers you give before any analysis takes place and will be deleted as soon as the study finishes. Your email address will not be passed to any third parties.

Your anonymised data will be stored in a password-protected file and may be used in academic publications. Because data is anonymised it will not be possible to withdraw your answers after they have been submitted but you can withdraw from future surveys at any point. Your IP address will not be stored. Research data will be stored for a minimum of three years after publication or public release of the findings of the research.

Because the data will all be anonymised we will not be able to act upon any individual responses to the survey.

Who will have access to my data?

The University of East London is ‘the data controller’ with respect to your personal data, and so will decide how your personal data is used in the study. The University will process your personal data for the purpose of the research outlined above. Research is a task that we perform in the public interest. Further information about your rights with respect to your personal data is available from https://www.uel.ac.uk/about/about-uel/governance/information-assurance/data-protection

We would also like your permission to use your anonymised data in future studies, and to share data with other researchers (e.g. in online databases). Any personal information (your email address) that could identify you will be removed or changed before files are shared with other researchers or results are made public.

Responsible members of the University of East London and funders may be given access to data for monitoring and/or audit of the study to ensure we are complying with guidelines, or as otherwise required by law.

The Principal Researcher is Dr Darren Sharpe, who is attached to the Institute of Health and Human Development at the University of East London.

d.sharpe@uel.ac.uk

This project has been reviewed by, and received ethics clearance through, the University of East London Research Ethics Committee **ETH1920-0212**.

University Research Ethics Committee

If you have any concerns regarding the conduct of the research in which you are being asked to participate, please contact:

Catherine Hitchens, Research Integrity and Ethics Manager, Graduate School, EB 1.43

University of East London, Docklands Campus, London E16 2RD

(Telephone: 020 8223 6683, Email: researchethics@uel.ac.uk)

1. If you have read the information above and agree to participate with the understanding that the data you submit will be processed accordingly, please click the box below to get started. ✱ *Required*

- ○ Yes, I agree to take part

Page 2: Socio Demographics

2. **Profile No.**

3. Gender:

- ☐ Male
- ☐ Female
- ☐ Prefer not to say

4. Age:

5. Nationality:

More info

- ○ Zambian
- ○ Sierra Leone

5.a. If you selected Other, please specify:

6. Sierra Leone Provinces:

7. Zambia Provinces:

8. Educational Level:

9. Type of disabilities:

10. Family Income/occupation per month:

Please don't select more than 1 answer(s) per row.

Please select at least 1 answer(s).

	Very low	Low	Medium	High	Very High	
0 $	☐	☐	☐	☐	☐	1,3600 $

11. Who owns land/housing:

11.a. If you selected Other, please specify:

Page 3: Wellbeing

The following block of questions is about thoughts and feelings. For each of the following statements,please select the option that best describes your experience ***over the last two weeks***.

12. I've been feeling optimistic about the future.

- None of the time (1)
- Rarely (2)
- Some of the time (3)
- Often (4)
- All of the time (5)

13. I’ve been feeling useful.

- None of the time (1)
- Rarely (2)
- Some of the time (3)
- Often (4)
- All of the time (5)

14. I’ve been feeling relaxed

- None of the time (1)
- Rarely (2)
- Some of the time (3)
- Often (4)

- All of the time (5)

15. I’ve been dealing with problems well.

- None of the time (1)
- Rarely (2)
- Some of the time (3)
- Often (4)
- All of the time (5)

16. I’ve been thinking clearly.

- None of the time (1)
- Rarely (2)
- Some of the time (3)
- Often (4)
- All of the time (5)

17. I’ve been feeling close to other people.

- None of the time (1)
- Rarely (2)
- Some of the time (3)
- Often (4)
- All of the time (5)

18. I’ve been able to make up my own mind about things.

- None of the time (1)
- Rarely (2)
- Some of the time (3)
- Often (4)
- All of the time (5)

Page 4: Social Networks

19. I feel I have lots of close friends who support me. ✱ *Required*

- None of the time
- Rarely
- Some of the time
- Often
- All of time

20. I have a lot of friends and family around me who I can trust.

- None of the time
- Rarely
- Some of the time
- Often
- All of time

21. Since the outbreak of COVID-19, I have felt more connected to others.

- None of the time
- Rarely
- Some of the time
- Often
- All of time

Page 5: My Response to COVID-19

22. We'd like to know how many people live in your household. Please tell us how many people, including yourself, living with you:

- I'm living alone
- 2 people
- 3 people
- 4 people
- 5 people
- 6 people or more

23. How many rooms are in your home? (Not including any bathrooms or toilets)

- 1 room
- 2 rooms
- 3 rooms
- 4 rooms
- 5 rooms
- 6 rooms or more

24. Do you have access to outside space where you can currently play, walk or hang out?

- Yes, in public places
- Yes, in my private space
- No

25. How would you rate your understanding to stay safe and well due to COVID-19 (Coronavirus)?

- Very poor
- Poor
- Moderate
- High
- Very High

26. Have you or other members of your family had COVID-19 (Coronavirus)?

- Yes, diagnosed and recovered well
- Yes, diagnosed and still ill
- Suspected corona-infected and still ill
- No

27. Are you worried about the long-term impact that COVID-19 (Coronavirus) and lockdown will have on your education training and work?

- Not at all
- Only a little
- Quite a lot
- A great deal

28. Which statement apply for you during epidemics of COVID -19 (Coronavirus)?

- I am living my life as normal and I don't care about others
- I am self-isolating as I am worried about spreading it to others

- I am self-isolating to protect my family members, friends who has an existing medical condition
- I am self-isolating because I am not allowed to go out

29. Have you or any member of your family received any help response towards Covid-19?

- Yes
- No

29.a. If yes, state the kind of help:

30. Do you feel you would need to be supported in relation to your response to COVID-19 and thereafter?

- Not at all
- Yes, a bit
- Yes, a lot
- Yes, completely

31. What kind of supports would you like receive in relation to your response to COVID-19 (Coronavirus) and isolation during epidemics and thereafter?

- [ ] Online written booklet and brochures about managing my emotions and behaviour
- [ ] Workshops, support group or seminars by professions
- [ ] Personalised online support from a professional or, charities or volunteer sectors

- [ ] Radio and TV programmes and podcasts
- [ ] Online helpline
- [ ] Other

31.a. If you selected Other, please specify:

32. Have you exercised during social distancing and taken part in sport?

- ( ) Not at all
- ( ) No more than usual
- ( ) Rather more than usual
- ( ) Much more than usual

33. Is there anything else you would like to add about how COVID-19 has impacted on you and your life?

34. Suggest ways in which sports can be used as a solution to Covid-19:

35. Interviewer initials:

36. Interview site:

37. Date of completion:

38. ID number:

Key for selection options

4 - Age:

12-14
15-17
18-20
21-23
24-25

6 - Sierra Leone Provinces:

Eastern Province

Northern Province
Southern Province
North West Province

7 - Zambia Provinces:

Central Province
Copperbelt Province
Eastern Province
Luapula Province
Lusaka Province
Muchinga Province
Northern Province
North-Western Province
Southern Province
Western Province

8 - Educational Level:

Primary Schooling
Secondary Schooling
College
University
Other

9 - Type of disabilities:

Physical Disabilities
Visual Disabilities
Hearing Disabilities
Mental Health Disabilities
Intellectual Disabilities
Learning Disabilities
two or more disabilities
Prefer not to say

11 - Who owns land/housing:

Mother
Father
Brother
Sister

Grandmother
N/A
Other
